# Lassa fever cases suffer from severe underreporting based on reported fatalities

**DOI:** 10.1093/inthealth/ihac076

**Published:** 2022-11-22

**Authors:** David Simons

**Affiliations:** Royal Veterinary College, Department of Pathology and Infectious Diseases, Centre for Emerging, Endemic and Exotic Diseases, 4 Royal College St., London, UK; London School of Hygiene and Tropical Medicine, Department of Clinical Research, Keppel Street, London, UK

## Abstract

**Background:**

Lassa fever is a viral haemorrhagic fever endemic to eight West African countries. Symptomatic disease is expected to occur in 20% of those infected and transmission typically occurs from viral spillover from rodent hosts. The combination of limited access to diagnostics and healthcare means the true burden of this disease is unknown.

**Methods:**

The case fatality rate among confirmed, probable and possible cases of Lassa fever in endemic regions is expected to be ≈15%. Here, annual reported cases and deaths have been used to estimate the case fatality rate, using three subsets of available data, to understand the scale of underreporting of severe human cases.

**Results:**

The literature review produced 38 records of cases and fatalities, comprising 5230 reported cases and 1482 reported deaths in seven countries. The estimated case fatality rate ranges from 16.5 to 25.6% (standard deviation 11.5–32.2). The expected number of severe cases between 2012 and 2022 is 8995, with current reported numbers 58% of what is expected.

**Conclusion:**

This analysis highlights current uncertainty and systemic underreporting of the morbidity and mortality burden of Lassa fever in its endemic region and must be considered when discussing the epidemiology of this neglected tropical disease.

## Introduction

Lassa fever, caused by *Lassa mammarenavirus*, is an endemic zoonotic infectious disease, with outbreaks of human infection regularly recorded in eight West African countries.^1^ Direct or indirect transmission from the primary zoonotic reservoir, the Natal multimammate mouse (Mastomys natalensis), is thought to be the source of most cases in endemic regions, with limited human-human transmission. Sporadic human cases are detected in non-endemic countries due to infected travellers. Most infections (≈80%) produce minimal symptoms, while symptomatic disease can lead to severe symptoms requiring hospitalisation and leading to death.

The number of individuals at risk of Lassa fever is projected to increase due to increasing human populations, land-use changes and climate change.^2^ Our understanding of the current impact across the endemic region is lacking due to limited diagnostics, surveillance and reporting. The degree of underreporting of cases presenting to healthcare is unknown, while the reporting of deaths associated with notifiable diseases such as Lassa fever is typically more complete. The case fatality rate (CFR) of Lassa fever is estimated at 15%, with wide variability. Two recent studies of hospitalised populations in Nigeria recorded CFRs of 14% and 31%, with a study in Sierra Leone estimating a CFR of 69%.^3–5^

The scale of underreporting can be estimated from the number of cases that would be expected to produce the number of reported deaths, under the assumption that these suffer from fewer limitations in reporting. The number of estimated cases can then be compared with the reported cases to produce a proportion of expected cases that are reported. This approach has been adopted during the current coronavirus disease 2019 (COVID-19) pandemic by organisations such as the World Health Organization (WHO) and can help to estimate the unrecognised burden of a disease.

## Methods

Reported Lassa fever cases were identified from a search of ProMED-mail, WHO Weekly Bulletin on Outbreaks and Other Emergencies, Nigeria Centre for Disease Control and Prevention (NCDC) situation reports and academic publications between 2012 and 2022. Where available, information on the number of suspected cases, confirmed cases and deaths among confirmed cases was extracted.

Three CFRs were calculated using the number of reported deaths as the numerator and cases as the denominator, weighted by the number of reported cases. First, across all reports obtained, if the number of deaths exceeded the number of confirmed cases, suspected cases were used as the denominator. Second, only NCDC data were used. This data includes prospective follow-up of confirmed cases and contact tracing, due to the impact of COVID-19 on healthcare-seeking only data prior to 2021 is included. Third, NCDC data limited to Edo and Ondo states between 2017 and 2021. The expected number of cases was calculated for reported deaths and compared with the number of reported cases. CFR values of 0% and 100% were removed prior to calculating weighted mean CFRs.

## Results

**Figure 1.**
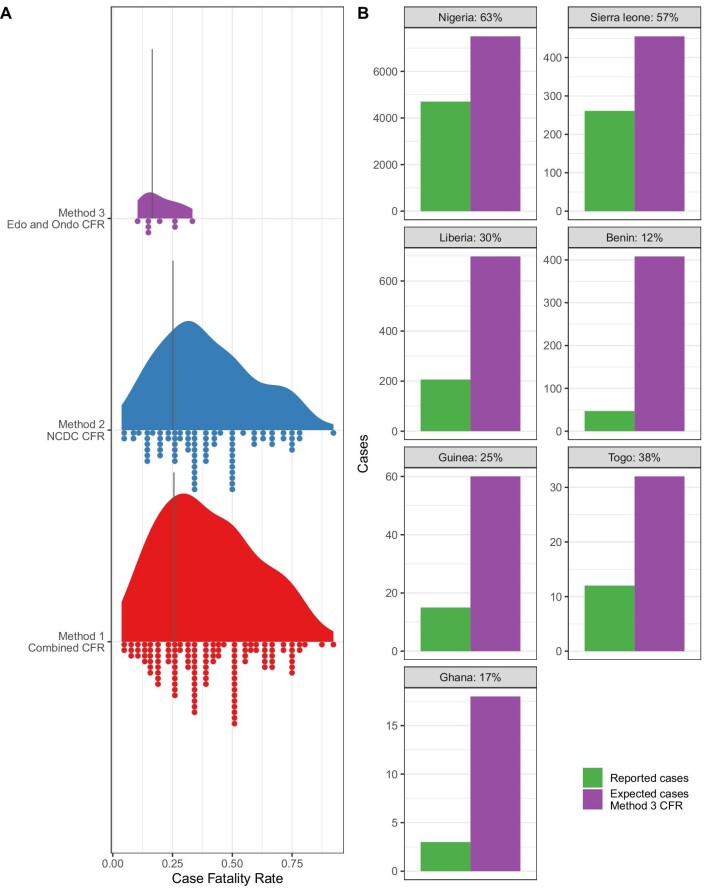
**(A)** CFR of Lassa fever following development of symptomatic disease and presenting to healthcare using three data sources for estimation. Method 1 uses all reported cases and deaths where the CFR is not equal to 0% or 100%. Method 2 uses all reported cases and deaths provided by NCDC data prior to 2021 where the CFR is not equal to 0% or 100%. Method 3 uses all reported cases and deaths from Edo and Ondo states from 2017 to 2021. The black line represents the weighted mean CFR. **(B)** The difference between reported cases and expected cases derived from the number of reported deaths divided by the CFR (note that the *y*-axis scale varies by country).

The literature review produced 38 records of cases and fatalities from seven countries between 2012 and 2022. These included 5230 reported cases and 1482 reported deaths. A similar CFR was estimated using the first two approaches (method 1: mean = 25.6% [standard deviation {SD} 16.6%]; method 2: mean = 25.2% [SD 16.2%]). Limiting the Nigerian states contributing data to those with higher surveillance (method 3) resulted in an estimated CFR of 16.5% (SD 5%; (Figure 1A). For the years 2018–2022, the number of reported cases from Nigeria was greater than the expected cases based on CFR estimates from methods 1 and 2, suggestive that a CFR of 16.5% (±5%) using method 3 is more representative of mortality following development of clinically severe disease. Estimates of CFR from method 3 show less variability than those including all outbreaks or all states, leading to greater confidence in this estimate. Applying this method of case estimation to other settings based on reported deaths found that between 17 and 63% of expected cases are reported (Figure 1B).

As expected, underreporting is greatest in countries in which Lassa fever surveillance is not routine and there are few reported deaths, i.e. Ghana, Guinea and Togo (17%, 25% and 38%, respectively). Conversely, in Nigeria and Sierra Leone, where surveillance is greater, underreporting was estimated at 63% and 57%, respectively. The lowest proportion of expected cases was reported from Benin (12%), which reports sporadic outbreaks based on identified deaths but has no routine surveillance. During the last decade, 5230 cases of Lassa fever have been reported, with 8995 expected cases, and with an estimated 3765 unreported cases.

These results are sensitive to the number of reported deaths due to Lassa fever, which is likely to suffer from variable reporting by country. As deaths are associated with individuals who present to clinical settings following symptoms, this method is unable to estimate the absolute number of cases in a given community. The CFR of Lassa fever has been treated as spatially non-varying, while the impacts of the known different viral strains on disease severity are currently unknown.

## Conclusions

The number of observed cases of Lassa fever is significantly underreported. This analysis has been performed to draw attention to the limitations of using reported case numbers when estimating the risk of disease in endemic countries and the risk of cases being exported from endemic countries.

## Data Availability

All data are available from open access sources. Analysis code and data to reproduce this analysis are available from https://github.com/DidDrog11/lassa_underreporting. Publication sources for included data are included in the supplemental dataset and at the above repository.
